# Chronic allergic lung inflammation negatively influences neurobehavioral outcomes in mice

**DOI:** 10.1186/s12974-022-02575-y

**Published:** 2022-08-31

**Authors:** Akihiro Kanaya, Mu Yang, Charles Emala, Maya Mikami

**Affiliations:** 1 Department of Anesthesiology, Vagelos College of Physicians and Surgeons, Columbia University, 630 West 168Th Street, P&S Box 46, New York, NY 10032 USA; 2 Department of Anesthesiology and Perioperative Medicine, Tohoku University Graduate School of Medicine, 2–1, Seiryo-Machi, Aoba-Ku, Sendai, 980-8574 Japan; 3 Mouse Neurobehavior Core, Institute for Genomic Medicine, Columbia University Irving Medical Center, 650 West 168Th Street, Black Building 1930, New York, NY 10032 USA

**Keywords:** Asthma, Chronic allergic lung inflammation, Depression, Spatial memory, Mast cell, Neurobehavior

## Abstract

**Background:**

Asthma is a major public health problem worldwide. Emerging data from epidemiological studies show that allergies and allergic diseases may be linked to anxiety, depression and cognitive decline. However, little is known about the effect of asthma, an allergic lung inflammation, on cognitive decline/behavioral changes. Therefore, we investigated the hypothesis that allergic lung inflammation causes inflammation in the brain and leads to neurobehavioral changes in mice.

**Methods:**

Wild-type C57BL/6J female mice were sensitized with nasal house dust mite (HDM) antigen or control PBS for 6 weeks to induce chronic allergic lung inflammation. A series of neurocognitive tests for anxiety and/or depression were performed before and after the intranasal HDM administration. After the behavior tests, tissues were harvested to measure inflammation in the lungs and the brains.

**Results:**

HDM-treated mice exhibited significantly increased immobility times during tail suspension tests and significantly decreased sucrose preference compared with PBS controls, suggesting a more depressed and anhedonia phenotype. Spatial memory impairment was also observed in HDM-treated mice when assessed by the Y-maze novel arm tests. Development of lung inflammation after 6 weeks of HDM administration was confirmed by histology, bronchoalveolar lavage (BAL) cell count and lung cytokine measurements. Serum pro-inflammatory cytokines and Th2-related cytokines levels were elevated in HDM-sensitized mice. In the brain, the chemokine fractalkine was increased in the HDM group. The c-Fos protein, a marker for neuronal activity, Glial Fibrillary Acidic Protein (GFAP) and chymase, a serine protease from mast cells, were increased in the brains from mice in HDM group. Chymase expression in the brain was negatively correlated with the results of sucrose preference rate in individual mice.

**Conclusions:**

6 weeks of intranasal HDM administration in mice to mimic the chronic status of lung inflammation in asthma, caused significant inflammatory histological changes in the lungs, and several behavioral changes consistent with depression and altered spatial memory. Chymase and c-Fos proteins were increased in the brain from HDM-treated mice, suggesting links between lung inflammation and brain mast cell activation, which could be responsible for depression-like behavior.

**Supplementary Information:**

The online version contains supplementary material available at 10.1186/s12974-022-02575-y.

## Introduction

Asthma, a chronic respiratory disease with airway inflammation and narrowing, is a major public health problem worldwide. Over 25 million people in the United States suffer from asthma, and allergic asthma is the most common subtype affecting ~ 60% of patients with asthma and more than 50% of patients with severe asthma [1, 2]. Emerging data from epidemiological studies show that allergies and allergic diseases may be linked to anxiety and/or depression in both adults and children [3–6]. An association between lung diseases and cognitive decline/dementia is also suggested [7]. Moreover, impaired learning and hippocampal changes were shown in an ovalbumin- induced mouse model of asthma [8].

Cognitive decline is often observed following surgery and during acute illness, events that are associated with systemic inflammatory responses that are known to disrupt the function of multiple organs [9, 10]. The primary pro-inflammatory cytokines involved in cognitive decline or behavioral changes that are characteristic of illness (i.e., loss of appetite, reduction in social behavior) are IL-1β and TNF-α [11]. These studies suggest that chronic systemic inflammation and the resultant increases in pro-inflammatory cytokines may affect specific brain functions; however, the effects of acute or chronic peripheral lung inflammation on brain functions and neurobehavioral outcomes are not well understood. An inflammatory reaction in the lungs involving cytokine release and/or immune cell activation following allergen exposure could disrupt the blood brain barrier, leading to inflammation in the brain and negatively influencing neurological functions. Alternatively, there is emerging evidence that there are bidirectional neuronal communications that could coordinate peripheral and central inflammation [12].

Patients with allergic asthma have increased levels of serum TNF-α [13, 14], which is known to disrupt the blood brain barrier [15]. Mast cells, which play key roles in allergic inflammation [16], are also found along cerebral blood vessels and contribute to brain inflammatory responses [17]. Whether allergic diseases and these well-characterized peripheral inflammatory events actually cause anxiety and/or depression is still not clearly demonstrated. Although the ovalbumin-induced mouse model of asthma is widely used to study allergic airway inflammation, using antigen models that more closely mimic human asthma would be optimal. House dust mites (HDM) are a significant source of indoor allergens, and they are also the prime cause of respiratory allergies including allergic rhinitis and allergic asthma. As many as 50% of asthmatic patients are sensitized with HDM [18]. Using an HDM mouse model, our group previously showed that 10 days to 3 weeks of intranasal HDM administration causes significant lung inflammation demonstrated by histological changes, increased cell counts in bronchoalveolar lavages (BAL), and increased protein concentrations in BAL as well as increased IL-1β and TNF-α in lung digests [19]. By extending the duration of HDM sensitization to mimic chronic states of allergic lung inflammation, we sought to test our hypothesis that chronic allergic lung inflammation causes inflammation in the brain, likely through cytokine-mediated disruption of the blood brain barrier, causing mast cell activation, resulting in neurobehavioral changes in mice.

## Methods

### Animals

**Fig. 1:**
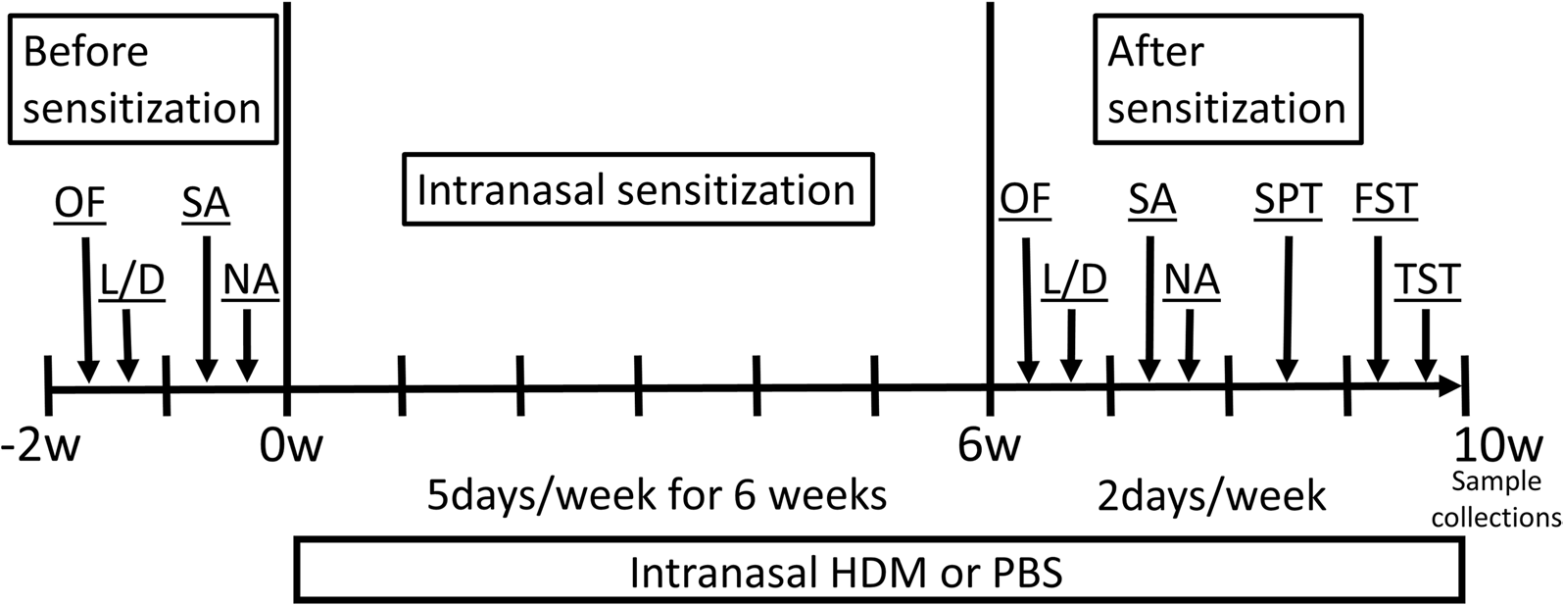
Schematic representation of the timeline of experiments. Before sensitization, OF, L/D, SA and NA were performed. Mice were sensitized by intranasal administration of house dust mite (HDM) or control PBS, 5 days/wk for 6 weeks. After sensitization, mice underwent behavioral testing using the OF, L/D, SA, NA, FST, TST and Sucrose preference test. The interval between each behavioral test was 2–3 days. 1. OF; open field, 2. L/D; light/dark transition test, 3. SA; Y-maze spontaneous alternation test, 4. NA; Y-maze novel arm test, 5. FST; forced swim test, 6. TST; tail suspension test. 7. SPT; sucrose preference test

All animal studies were approved by the Columbia University Institutional Animal Care and Use Committee. 8- to 10-week-old C57BL/6J female mice were purchased from the Jackson Laboratory (Bar Harbor, ME, USA) and allowed to acclimate to the vivarium prior to the experiments. All animals were initially housed in groups of four mice per cage. Only females were used because we observed constant fighting in group-housed males in a previous pilot study which would alter behavioral studies. The timeline of experiments is shown in Fig. 1. House dust mite (HDM) extracts were purchased from Stallergenes Greer (Lenoir, NC, USA) and reconstituted in endotoxin-free PBS (MilliporeSigma, Burlington, MA, USA) and 30 μg (in 25 μL) were administered intranasally under brief 2–5% isoflurane anesthesia, once daily for 5 days a week for 6 weeks. Behavioral experiments commenced at this time point over an additional 4 weeks during which time HDM or PBS treatments were continued for 2–3 times each week. PBS control mice received the same volume of PBS intranasally under the same anesthesia protocol.

### Behavioral experiments

A battery of behavioral tests were carried out to evaluate locomotion, as well as anxiety-like and depressive-like behaviors before and after intranasal HDM administration. The mice were divided into two groups for the HDM sensitization as follows: (i) Control group (*n* = 12 PBS group); (ii) HDM group (*n* = 12 HDM group). Open field (OF), light and dark box (L/D), Y-maze spontaneous alternation (SA) and Y-maze novel arm (NA) tests were performed for baseline measurements during a 2-week period before HDM sensitization or control PBS exposure (Fig. 1). After the 6 weeks of intranasal PBS or HDM administration these tests (OF, L/D, Y-maze SA, Y-maze NA) plus additional tests (forced swim test (FST), tail suspension test (TST) and sucrose preference test) were performed over a 4-week period (during continued HDM or PBS exposure) to evaluate anxiety-like and depressive-like behaviors (Fig. 1).

#### Open field test

The open field test was performed following a previously described protocol [20]. Exploration was monitored during a 60-min session with Activity Monitor Version 7 tracking software (Med Associates Inc., Fairfax, VT, USA). Each mouse was gently placed in the center of a clear plexiglass arena (27.31 × 27.31 × 20.32 cm, Med Associates ENV-510) lit with dim light (~ 5 lx), and was allowed to freely explore the chamber for the duration of the trial. Infrared (IR) beams embedded along the *X*, *Y*, *Z* axes of the arena automatically tracked the distance moved, horizontal movement, vertical movement, stereotypies, and the time spent in the center zone (14.29 × 14.29 cm). Data were analyzed in six, 10-min time bins. Arenas were cleaned with 70% ethanol and thoroughly dried between trials.

#### Light–dark transition test

This test examines anxiety-like behavior in rodents. The test was conducted for 10 min in a rectangular box (60 × 30 × 30 cm). The chamber was divided into two compartments, a light (~ 350 lx) and a dark side (< 5 lx), with a transition zone (door) that allows the mouse to cross between the two compartments. At the start of the trial, the mouse was placed in the light side and allowed to ambulate freely for the entire duration of the trial. The number of transitions and times spent in the light vs. dark zones were recorded by a camera mounted above the light and dark box that was interfaced with the Ethovision XT 12 software (Noldus Information Technology Inc., Leesburg, VA, USA) for analysis.

#### Y-maze test

The Y-maze measures exploratory behavior and can be used to assess spatial memory and working memory in mice. The test was conducted in the Y-maze apparatus (Maze Engineer, Skokie, IL, USA), which consists of three arms of equal length (35 cm), arm lane width (5 cm), and wall height (10 cm). A camera mounted above the maze and interfaced with the Ethovision XT 12 software (Noldus Information Technology Inc.) automatically records the distance traveled, arm entries, and the time spent in each arm. The Y-maze spontaneous alternation is a one 8-min trial in which the subject was allowed to freely explore all three arms. The mouse usually begins to perform alternations, otherwise known as entering a new arm each time it moves. The percent of alternations were analyzed as a measure of working memory.

In the Y-maze novel arm preference test, a 2 cm × 2 cm sticker (with an image of an equal sign, a bus or a plane) is taped at the end of each lane, one inch above the floor. The start arm was always marked with the equal sign, and the bus and the plane stickers were randomly assigned to the familiar or the novel arm. In trial 1, each mouse was placed in the start arm and allowed access to the start arm and one other arm (the familiar arm) for a 10-min session. A removable opaque door blocked access to the third arm. At the conclusion of trial 1, the mouse was placed in a temporary holding cage for 10 min. In the memory test (Trial 2), the opaque door was removed, and the mouse was returned to the start location, now free to explore all three arms for 5 min. The designation of the novel arm and familiar arm was randomly assigned and changed across animals. Preference scores were calculated as, the time spent in the novel arm/(time spent in the novel arm + time spent in the familiar arm) × 100.

#### Tail suspension test (TST)

The TST is widely used to measure depressive-like behavior along with the FST. The TST was performed for a 6‐min test period. Each individual mouse was suspended about 30 cm above the floor by adhesive tape placed ~ 1 cm from the tip of the tail. The activities of the mice were videotaped, and we calculated the time of immobility during a 6-min testing period. In order to avoid climbing on its own tail, cylinders (4 cm length, 1.6 cm outside diameter, 1.3 cm inside diameter, 1.5 g) were placed around the tails of mice.

#### Forced swim test (FST)

Mice were individually placed in a cylinder (20 cm diameter, 40 cm height) containing 30 cm (depth) of water at 22–24 °C and allowed to swim for 6 min. The water depth was set to prevent mice from touching the bottom of the cylinder with tails or hind limbs. The activities of the mice were videotaped, and we calculated the time of immobility during the 6-min testing period. The mouse was judged to be immobile when it floated motionless in the water with necessary small movements to keep its head above the water.

#### Sucrose preference test

The two-bottle choice test is a standard assay for testing anhedonia and depressive-like behaviors. During the baseline exposure period, each mouse had access to regular chow and two bottles (Nalgene 50-ml centrifuge tubes, Nalge Nunc International Corporation, Rochester, NY, USA) in their home cage, one containing 1% sucrose and the other one containing regular tap water, for 48 h, with the position of the two bottles changed every 24 h to avoid bottle location bias. Then each mouse was moved to its own cage and given just regular tap water (from one standard water bottle) and regular chow for the next 24 h. On the testing day (8 pm to 8 am), mice were again given two bottles, one with fresh 1% sucrose solution and the other one with fresh tap water in a single cage. At the end of the test, both bottles were collected, and the contents weighed. During the two-bottle choice test, regular bedding was removed and replaced with few pieces of paper towels in order to detect any potential bottle leakage. Nalgene tubes were washed with fragrance free detergent (Seventh Generation, Burlington, VT, USA) and air-dried after each use.

### Bronchoalveolar lavage (BAL) cell count and protein concentration measurement

Following the completion of all behavioral tests (~ 10 week time point, Fig. 1), mice were anesthetized with an intraperitoneal injection of pentobarbital sodium (100 mg/kg), and tracheotomized with an 18 g metal cannula. BAL fluids were collected by slow injections and aspirations of 1 ml PBS through the cannula. This was repeated a second time and the two aliquots were combined. BAL cell suspensions were centrifuged at 200×*g* for 5 min and the resulting BAL supernatant were transferred to the new tube. The pellets were resuspended in PBS and the cell numbers were manually counted using a hemocytometer. Protein concentrations in the BAL supernatant were measured using the Pierce BCA protein assay kit (Thermo Fisher Scientific, Waltham, MA, USA).

### Cytokine assay

Whole blood samples were collected by direct cardiac puncture. They were allowed to clot for 2 h at room temperature and the sera were collected after centrifugation (2000×*g* for 20 min at room temperature) which were stored at − 80 °C. After whole body perfusion with PBS via the cardiac puncture, dissected whole left lungs and half brains were snap-frozen in liquid nitrogen. Later they were lysed using a motorized tissue cutter in lysis buffer containing 0.5% Nonidet P40, 150 mM NaCl, 20 mM Tris (pH 7.5) and freshly added protease inhibitor cocktail (cOmplete, MilliporeSigma). Lysed samples were centrifuged at 5000×*g* for 15 min at 4 °C, and the supernatants were collected, and their protein concentrations were measured using Pierce BCA protein assay kit (Thermo Fisher Scientific). The serum, lung and brain levels of cytokines and chemokines were measured by Eve Technologies (Calgary, AB, Canada) using a multiplex Mouse Cytokine Array.

### Flow cytometry

After perfusion with PBS, dissected right lung lobes and half brains were rinsed in ice-cold PBS, then minced with a curved scissor. Small pieces of lung tissue were incubated in pre-warmed digestion buffer containing 1 mg/mL type IV collagenase, 1 mg/mL soybean trypsin inhibitor, 0.1 mg/mL DNAse I in RPMI medium 1640 (Gibco, Thermo Fisher Scientific) for 40 min at 37 °C with gentle agitation. The suspension was minced through a 40-µm cell strainer, and the resulting cells were collected after centrifugation (200×*g* for 5 min), washed in RPMI medium 1640 and the cell numbers counted using a hemocytometer. Small pieces of brain tissue were minced through a 100-µm cell strainer, rinsed in ice-cold HBSS and centrifuged at 286×*g* at 4 °C for 5 min. The cell pellet was resuspended in pre-warmed digestion buffer containing 0.4 units/mL Liberase TL, 0.1 mg/mL DNAse I in HBSS with Ca^2+^ and Mg^2+^ and incubated under slow continuous rotation at 37 °C for 1 h. The brain cell suspension was sieved through a 70-µm cell strainer, rinsed with 10% fetal calf serum (FCS) in HBSS and centrifuged at 286 × g at RT for 5 min. The resulting pellet was resuspended in 25% Percoll (GE healthcare, Chicago, IL, USA) density gradient medium containing 3% FCS, and centrifuged at 520×*g* at 18 °C for 20 min. The brain cell pellet was washed two times to remove the density gradient medium. The collected lung and brain cells were stained overnight at 4 °C in 0.2% bovine serum albumin in PBS containing 1:100 dilution of the following antibodies from BD Bioscience (Franklin Lakes, NJ: CD3e-Pacific Blue (558214), CD45-APC (559864), CD117-PE-CF594 (562417) and FceR1α-BV510 (751757). Analytical cell counts were performed using a BD LSR II flow cytometer, and the data were analyzed using FCS Express 7 software (De Novo Software, Pasadena, CA, USA).

### Immunohistochemistry

Mice for immunohistochemistry experiments were subjected to whole body perfusion via a right heart catheter with PBS and then with 4% paraformaldehyde (0.1 M phosphate buffer, pH 7.4). The lungs were dissected out from chest cavity, and fixed in 10% formalin (Fisher Diagnostic, Thermo Fisher Scientific) for 24 h, dehydrated through serial ethanol concentrations, and embedded in paraffin. Lung slices (6 μm) were stained with hematoxylin and eosin. Images were obtained with an inverted microscope (Olympus IX-70, Tokyo, Japan). Dissected whole brains were fixed in 10% formalin for 48 h and paraffin embedded. 7 μm sections were dewaxed in SafeClear Xylene Substitutes (PROTOCOL, Thermo Fisher Scientific), and rehydrated in a graded alcohol series to water. Heat-mediated antigen retrieval was performed with sodium citrate buffer (10 mM, pH 6.0) for 10 min maintaining at a sub-boiling temperature and slides were cooled on the bench top for 30 min. Endogenous peroxidase was blocked using 0.3% hydrogen peroxide for 30 min at room temperature. Sections were blocked with 10% normal goat serum for 60 min. After sections were washed with PBS, an avidin biotin blocking kit (Vector Laboratories, Inc., Burlingame, CA, USA) was used to block endogenous biotin. Slides were rinsed with PBS containing 0.1% Triton X-100 (PBST) and incubated overnight at 4 °C in primary antibodies against the Iba-1 protein (rabbit polyclonal 1:100, ab178846, Abcam, Cambridge, UK), the c-Fos protein (rabbit polyclonal 1:100, ab190289, Abcam) or the chymase protein (rabbit polyclonal 1:100, ab233103, Abcam) in 2% normal goat serum in PBST. After overnight incubation at 4 °C, slides were washed three times with PBST, and primary antibodies were detected using biotinylated anti-rabbit antibodies (Vector Laboratories, Inc.) at a concentration of 1:100. After incubation with ABC-HRP complex (Vector Laboratories, Inc.) for 30 min, the antigen–antibody complex was then visualized with the DAB peroxidase substrate kit (Vector Laboratories, Inc.). Sections were dried, and coverslipped using Eukitt (MilliporeSigma).

### Western blotting

Western blotting was carried out by standard methods, and 75 μg samples of brain homogenate were subjected to SDS-polyacrylamide gel electrophoresis. Briefly, the half brain samples were lysed in an ice-cold lysis buffer (0.5% Nonidet P40, 150 mM NaCl, 20 mM Tris pH 7.5) containing freshly added protease inhibitor (cOmplete, MilliporeSigma) for 30 min on ice and sonicated (50% power for 2 s × 10 pulses, SONICS Vibra-Cell Ultrasonic processor, Sonics & Materials Inc., Newtown, CT, USA). Lysed samples were centrifuged at 5000×*g* for 15 min at 4 °C and the supernatant was combined with Laemmli’s sample buffer and incubated at 100 °C for 5 min before loading on a 4–15% Mini-PROTEAN TGX gel (Bio-Rad Laboratories, Inc., Hercules, CA, USA). Proteins were separated by electrophoresis and transferred to a 0.45 µm nitrocellulose membrane (Bio-Rad). The membrane was blocked in 3% milk–Tris buffered saline (50 mM Tris–HCl, pH 8.0, 100 mM NaCl) supplemented with 0.1% Tween 20 (TBST), probed with the following primary antibodies (all 1:2000 dilution): c-Fos (ab190289, Abcam), Iba-1 (ab178846, Abcam), chymase (ab233103, Abcam),GFAP (NB300-141, Novus Biologicals, Littleton, CO, USA), cleaved caspase-3 (9661, Cell Signaling, Danvers, MA, USA), or GAPDH (ab9784, Abcam) all in 1% milk–TBST, and reacted with the horseradish peroxidase-conjugated secondary antibody (1:2500) in 1% milk–TBST. After reaction with the Western chemiluminescence reagent (SuperSignal West Femto Maximum Sensitivity Substrate, Thermo Fisher Scientific), the images were captured on the Gel Doc XR + Gel Documentation System (Bio-Rad). Densitometric quantitation was performed using ImageJ software (National Institute of Health, USA).

### Statistical analysis

The data are presented as mean ± SEM and were analyzed using Graph Pad Prism software (GraphPad software, v8, Inc., La Jolla, CA, USA). Unless otherwise stated, Mann–Whitney or Wilcoxon paired t-tests were used to compare two groups: PBS control and HDM groups. Two-way repeated measures ANOVA was used to analyze open field data. One-way repeated measures ANOVA was used to compare the duration of the time spent in each arm during the Y-maze novel arm tests. A two-sided *p*-value of less than 0.05 was considered significant.

## Results

**Fig. 2:**
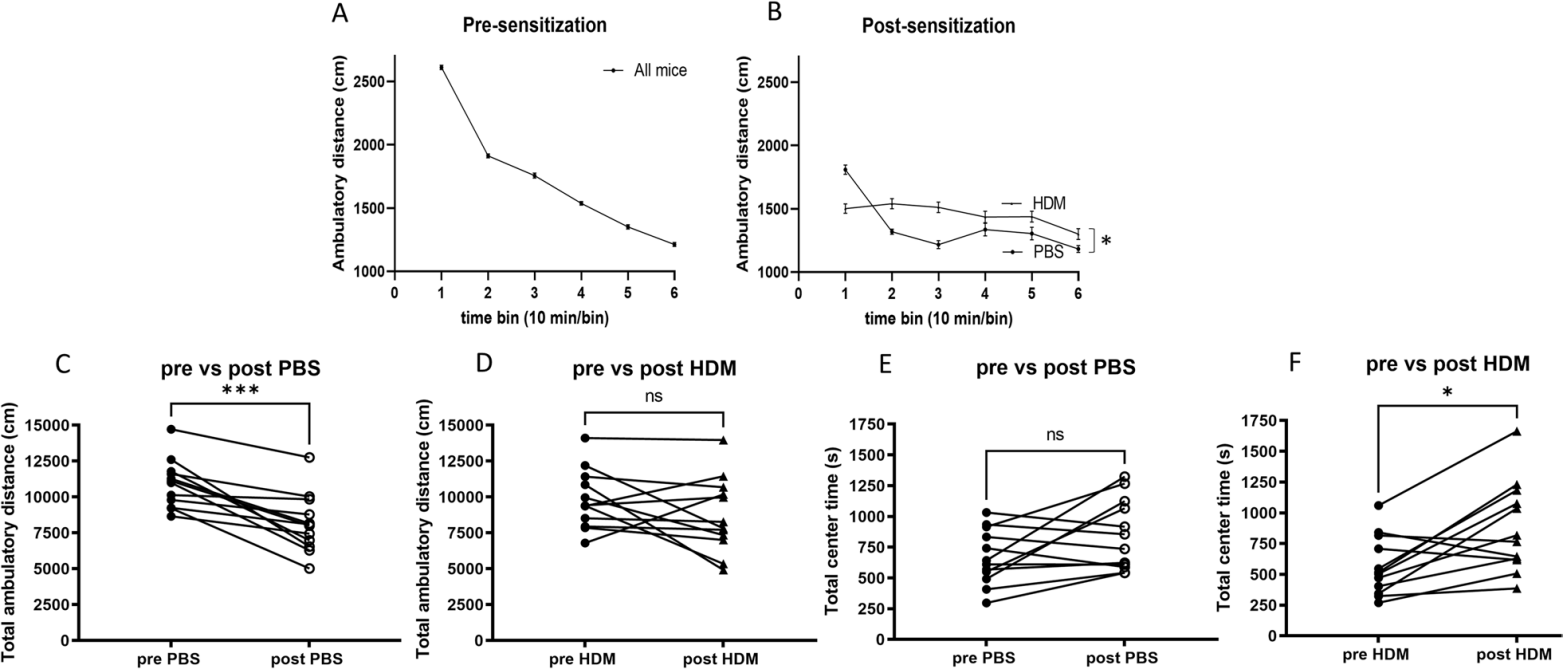
Locomotor activity measured by the open field test before and after 6 weeks of intranasal HDM or PBS (control). **A**, **B** The ambulatory distance over each 10-min time bin during the 60-min trial before (*n* = 24, total mice) and after (*n* = 12 per group) intranasal sensitization. *p < 0.05, comparing exploratory activity over the duration of the session, between treatment groups, two-way ANOVA. **C**, **D** A comparison of the total ambulatory distance in individual mice, pre- and post- intranasal sensitization with HDM or PBS (control). Mice in the HDM group did not show a typical reduction of ambulatory distances traveled after sensitization. **E**, **F** A comparison of the total time spent in the center of the open field chamber in individual mice (*n* = 12 each, *p < 0.05, ***p < 0.001, Wilcoxon paired *t*-test)

The Open Field test was performed to assess spontaneous locomotor activity, a reflection of novel environment exploration, as measured by the ambulatory distance, vertical count and the time spent in the center zone of the plexiglass arena. As expected, before exposures to control PBS or HDM treatments, no significant group differences were found on ambulatory distance in the 60-min trial. Both groups of mice ambulated less as time went on, reflecting habituation to the environment (Fig. 2A). After nasal HDM sensitization, this typical pattern of habituation was absent. In contrast, PBS control mice exhibited a normal pattern of habituation (Fig. 2B, *p* = 0.017). A comparison of the total ambulatory distance traveled by individual mice before vs after PBS or HDM treatments revealed a reduction in the traveled distance in the control PBS group (Fig. 2C, *p* = 0.0005). This reduction of ambulatory distance is typically seen in normal mice tested repeatedly in the same open field arena. However, this reduction was not observed in the HDM sensitization group (Fig. 2D, *p* = 0.1514). Vertical count reflects rearing behavior and was not different between the two groups (Additional file 1: Fig. S1A) after treatments with PBS or HDM, indicating similar general physical motor abilities. The amount of time spent in the center of an open field chamber was not different between the groups (Additional file 1: Fig. S1B). Reduced time spent in the center is usually interpreted as anxiety-like behavior. When comparing pre- versus post-PBS or HDM treatment center times for individual mice, we observed an increased center time in HDM-treated mice (Fig. 2F, *p* = 0.0122) but not in PBS control mice (Fig. 2E, *p* = 0.1514). Comparing mean center times or total ambulatory times across the groups after PBS or HDM treatment did not demonstrate a difference between the groups (Additional file 1: Fig. S1B, C). The increase in center time in individual mice comparing pre- and post-HDM treatment (Fig. 2F) is not due to immobility from the center location, because the group mean of the total ambulatory distance was not different between groups treated with PBS versus HDM (Additional file 1: Fig. S1C).

**Fig. 3:**
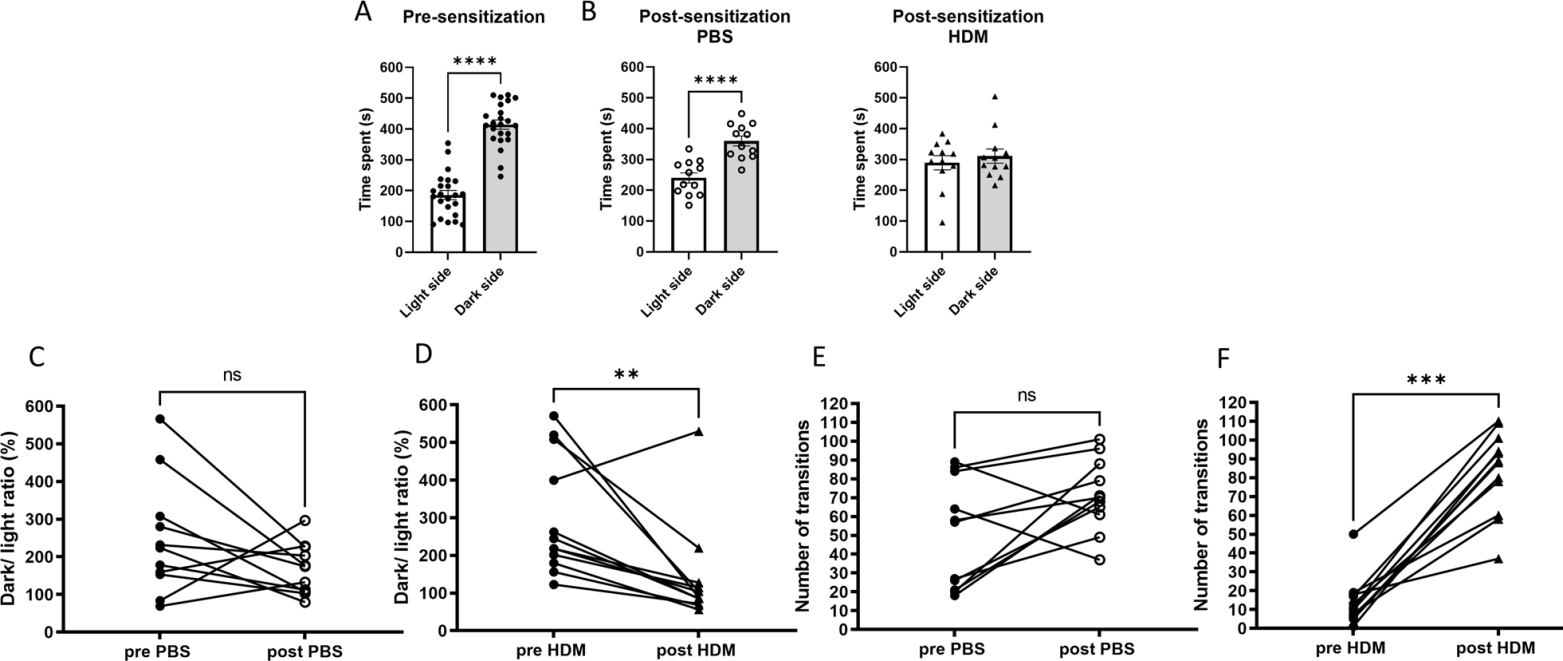
Exploratory behavior and the animal’s preference for the light measured in the light–dark transition test. **A** Before initiation of nasal PBS or HDM treatment, mice spent more time in the dark side. **B** After PBS treatment, control mice maintained a preference for the dark while HDM-sensitized mice spent about the same time in the light and dark chambers, which is unusual considering rodents’ natural aversion to bright spaces. ****p < 0.0001, Mann–Whitney. **C**, **D** The time ratio spent in the dark versus the light in individual mice before and after the sensitization was significantly lower in HDM group but not in the control PBS group. **p < 0.01, Wilcoxon paired *t*-test. **E**, **F** The number of transitions (how often the light–dark transition zone was crossed) in individual mice was remarkably higher in the HDM group after sensitization. ***p < 0.001, Wilcoxon paired *t*-test

The light–dark test was used to measure anxiety-like behaviors in mice, and the time spent in each chamber (dark or with light) and the number of transitions were counted. In this test, mice usually prefer to stay in the dark chamber, as we observed in pre-sensitized mice (Fig. 3A, *p* < 0.0001) and PBS control mice (Fig. 3B, *p* < 0.0001). Interestingly, HDM-treated mice spent about the same amount of time in the light and dark chambers (Fig. 3B, *p* = 0.8428), which is very unusual behavior considering the natural aversion of rodents to bright spaces. We also analyzed the ratio of the time duration that was spent in the dark chamber over the time spent in the light chamber and compared these times in the pre- and post-sensitization in individual mice in each group. This ratio significantly decreased in the HDM group (*p* = 0.0093) but not in the PBS control group (*p* = 0.1748) (Fig. 3C, D), indicating that HDM-treated mice had an increased preference to spend time in the light chamber. Rodents are known to display a tendency toward exploratory behavior which is measured by the number of transitions in the light–dark test. When the number of transitions were compared in individual mice before and after sensitization, there was no difference in the PBS control group (*p* = 0.0781). HDM-treated mice showed a remarkable increase in the transition frequency compared to the pre-HDM level (Fig. 3F, *p* = 0.0005). In general, an increase in transition frequency is interpreted as reflecting a reduction in anxiety-like behaviors.

**Fig. 4:**
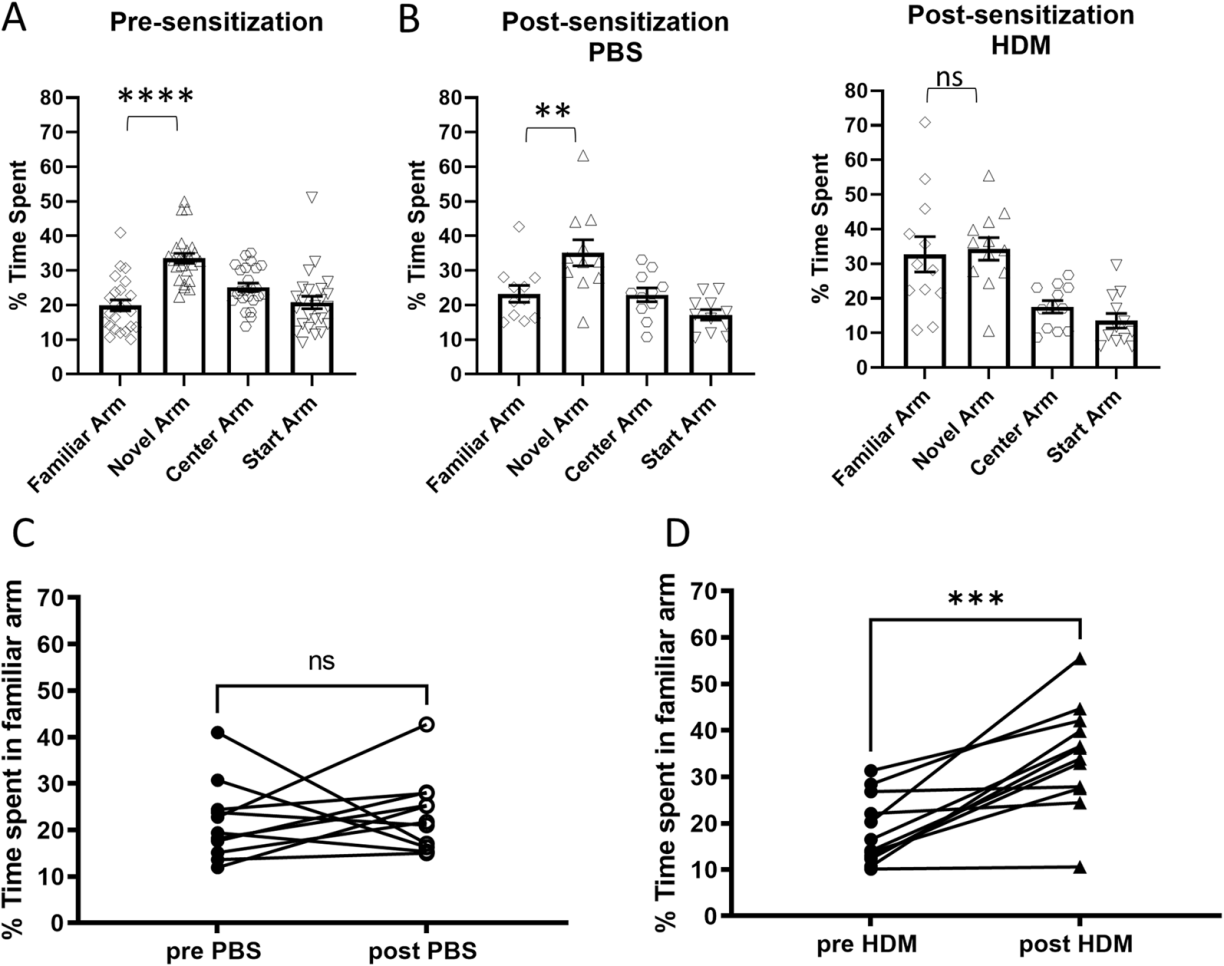
Spatial memory assessed by the Y-maze novel arm test. **A** Before sensitization, mice spent more time in the novel arm compared to the familiar arm. **B** After treatment, control mice again spent more time in the novel arm than the familiar arm, however, the HDM-sensitized mice spent about the same time in the familiar arm, indicating poor spatial memory. ****p < 0.0001, **p < 0.01, ns not significant, one-way ANOVA novel vs. familiar. **C**, **D** A comparison of the percent time spent in the familiar arm in individual mice. Only mice in the HDM group showed an increased time spent in the familiar arm, again indicating impaired spatial memory. ***p < 0.001, Wilcoxon paired *t*-test

The Y-maze novel arm test was used to assess spatial memory. Mice were placed in the starting arm, allowed to explore with one arm closed off by a door, and were then reintroduced to the maze with both the familiar and novel arms open. Generally, mice prefer to explore a novel arm and a new object as was observed in pre-sensitized mice (Fig. 4A, novel vs. familiar, *p* < 0.0001) and PBS control mice (Fig. 4B, left, novel vs. familiar, *p* = 0.0083), if they have normal spatial memory. However, after sensitization, HDM-treated mice spent more time in the familiar arm (Fig. 4B, right), indicating impaired spatial memory in this group. This observation was evident when individual mouse behavior was compared before and after sensitization. Compared with PBS control, mice sensitized with HDM spent significantly more time in the familiar arm (Fig. 4C, *p* = 0.5771, Fig. 4D, *p* = 0.0005, respectively).

**Fig. 5:**
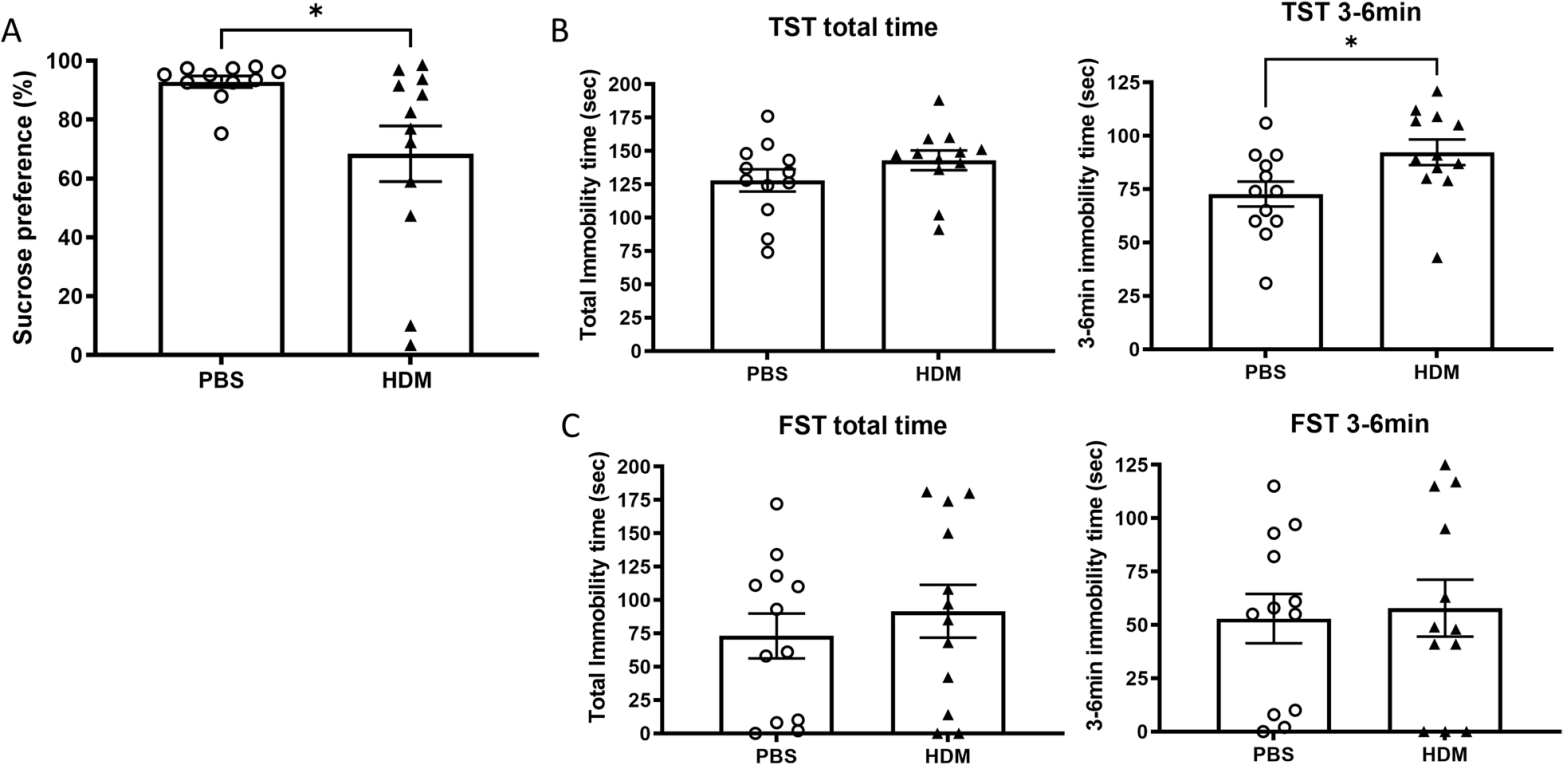
Depression-like behaviors assessed by the sucrose preference test, the tail suspension test and the forced swim test. **A** Mice treated with intranasal HDM exhibited decreased sucrose preference compared with PBS control mice. *p < 0.05, Mann–Whitney. **B** After 6 weeks of intranasal PBS (control) or HDM administration, mice underwent the tail suspension test (TST) and the duration of no movement (immobility time) was recorded. The HDM group showed increased immobility time during the last 3 min, indicating increased depressive-like behavior (*p < 0.05, Mann–Whitney). **C** In the forced swim test (FST), no significant difference was observed between mice in the PBS control versus HDM sensitization group

The sucrose preference test is a standard assay for testing anhedonia and depressive-like behaviors in rodents. In general, mice seek out a sweet rewarding drink relative to plain drinking water. PBS control mice showed the expected preference toward the sweetened drink; however, we found significantly reduced sucrose water consumption in HDM-treated mice (Fig. 5A, *p* = 0.0256), indicative of anhedonia and depression in this group. In the TST and FST, we calculated the immobility time during the last 3 min of the total 6-min test time to remove the effect of active phases at the beginning [21]. The TST enables us to measure depression-related behaviors, and has been used to screen potential antidepressant drugs [22]. Immobility times between the PBS group and HDM group were not significantly different during the total 6-min trial (*p* = 0.1037), however, during the last 3 min immobility times were longer in the HDM group compared to the PBS group (*p* = 0.0270) (Fig. 5B), suggesting changes in depression-like behaviors. The FST was also performed following sensitization to examine the long-term effects of chronic allergic lung inflammation on depression-like behavior [23]. Immobility times between the PBS group and HDM group in the FST were not significantly increased in during the total 6-min trial or during the final 3-6 min time periods (Fig. 5C, *p* = 0.589 and 0.4830, respectively).

**Fig. 6:**
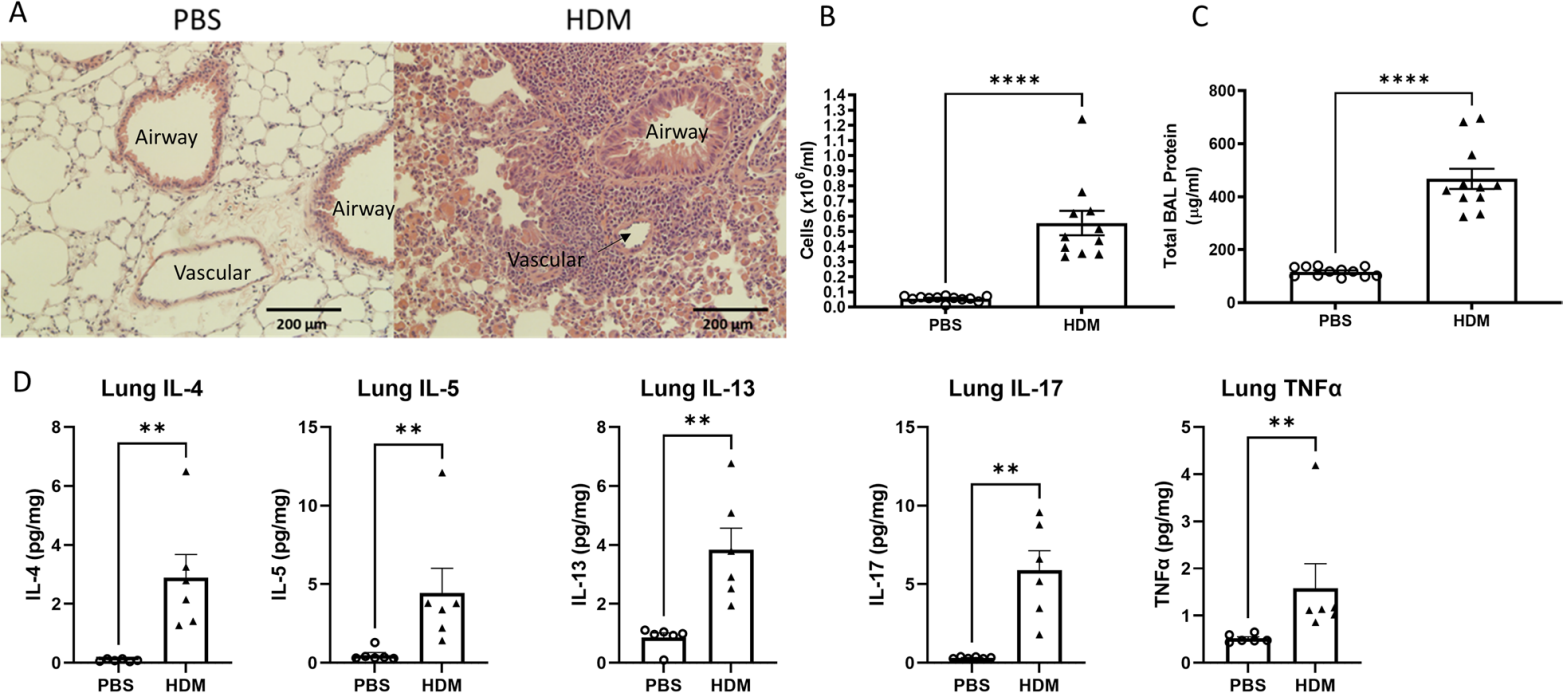
Six weeks of nasal HDM administration caused lung inflammation measured by histology, BAL cell counts, BAL total protein concentrations and lung cytokine measurements. **A** Six weeks of intranasal HDM administration caused marked peri-bronchial and peri-vascular infiltration of mononuclear cells. **B** Total cells counted in the bronchoalveolar lavage (BAL) were significantly higher in the HDM group. **C** Protein concentrations in the BAL were elevated in the HDM group. **D** Multiple pro-inflammatory cytokine concentrations in lung digests were higher in the HDM group compared to PBS control. **p < 0.01, ****p < 0.0001, Mann–Whitney

Next, we sought to confirm inflammation in the lungs after long-term intranasal HDM sensitization. We have previously demonstrated that 10 days to 3 weeks of intranasal HDM administration can cause significant lung inflammation as assessed by histological changes, bronchoalveolar lavage (BAL) cell count, BAL protein concentrations and cytokine measurements in lung digests [19]. In the current study, we extended the duration of HDM administration to 6 weeks to mimic the chronic status of lung inflammation. Some experiments, however, have demonstrated that long-term allergen exposure resulted in weakened airway responsiveness and remodeling [24], thus, we wanted to confirm whether the lung inflammation persisted beyond the typical 2–3 weeks of HDM sensitization. Hematoxylin and eosin staining of lung sections showed marked infiltration of mononuclear cells in the peri-vascular areas and around small airways after 6 weeks of intranasal HDM administration compared with the PBS control (Fig. 6A). Analysis of the BAL showed an increased number of total cells (Fig. 6B, *p* < 0.0001) and protein concentrations (Fig. 6C, *p* < 0.0001) in the HDM group compared with the PBS control group, confirming the presence of lung inflammation caused by HDM administration. Cytokines measured in the lung digest showed increased levels of Th2 cytokines (IL-4, 5, 13) (*p* = 0.0022), the Th17 cytokine IL-17 (*p* = 0.0022) and TNF-α (*p* = 0.0022), a pro-inflammatory cytokine implicated in airway pathology of asthma and potentially an important cytokine found in refractory asthma [25–27].

**Fig. 7:**
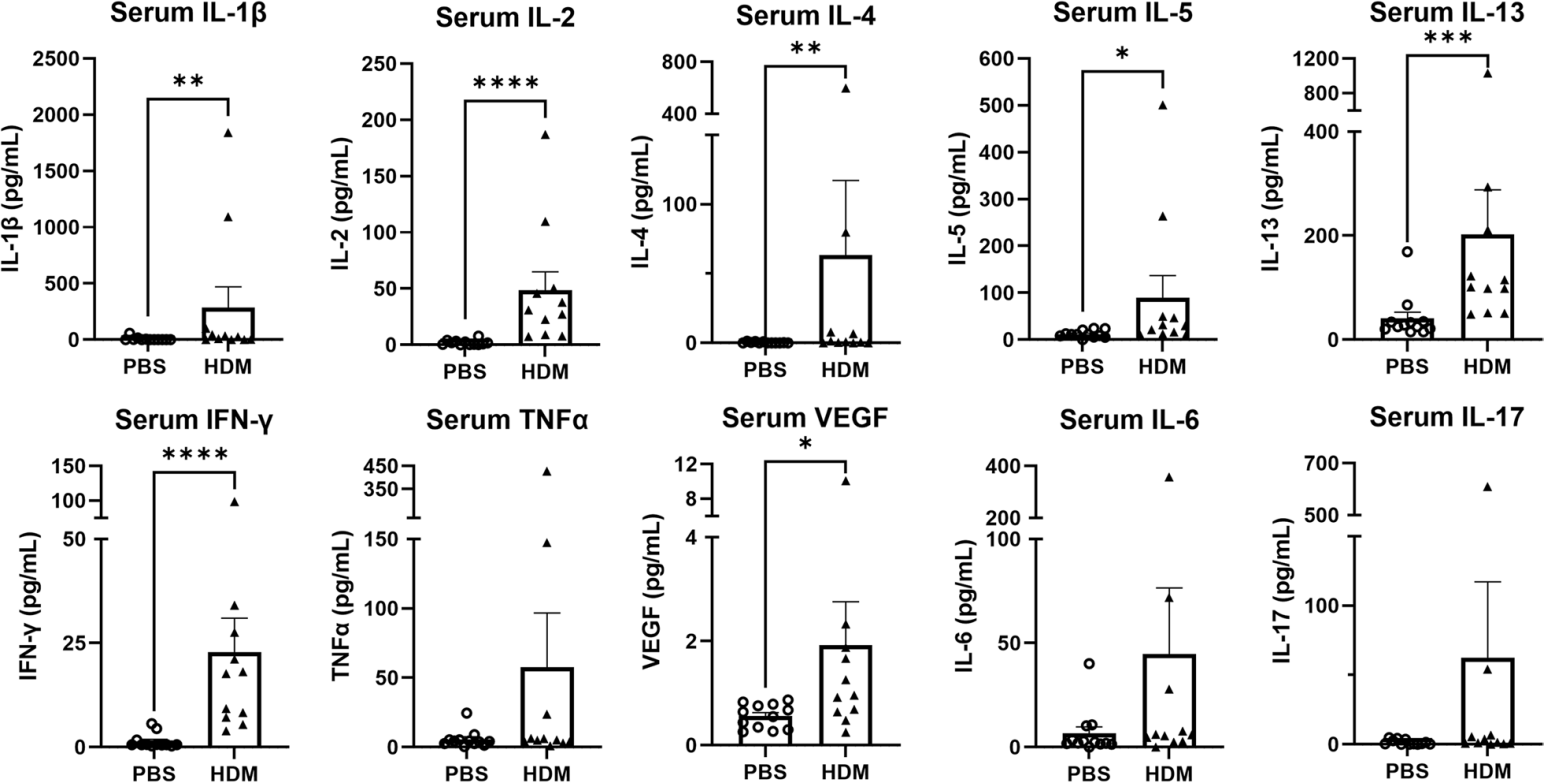
Cytokines measured in the serum after 6 weeks of nasal PBS or HDM sensitization. After chronic intranasal HDM sensitization, changes in serum cytokine concentrations were observed. *p < 0.05, **p < 0.01, ***p < 0.001, ****p < 0.0001, Mann–Whitney

To investigate whether chronic local lung inflammation leads to systemic changes in the levels of circulating cytokines, we measured a variety of serum cytokines. The levels of pro-inflammatory cytokines IL-1β, IL-2 and IFN-γ, as well as Th2-related cytokines IL-4, IL-5 and IL-13 were significantly elevated in the serum of HDM-sensitized mice compared to PBS control mice (Fig. 7), mirroring the Th2-mediated allergic inflammation demonstrated in the lungs after HDM administration. Serum vascular endothelial growth factor (VEGF) concentrations was significantly elevated in HDM-sensitized mice compared to control mice. Serum TNFα, IL-6 and IL-17 levels were not significantly different between the PBS control and HDM-sensitized groups (Fig. 7).

**Fig. 8:**
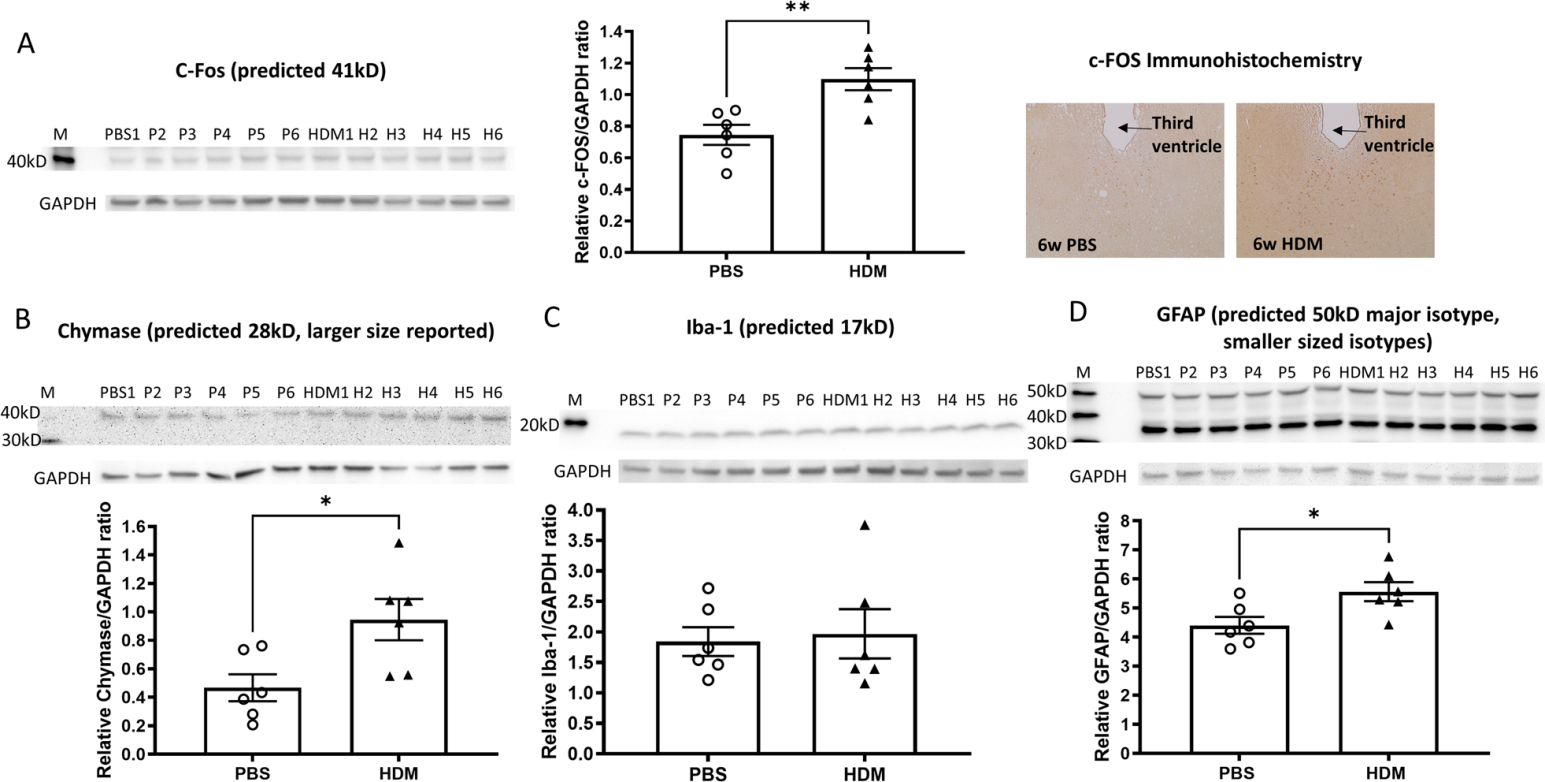
Brain pro-inflammatory and cell activation-associated protein levels. **A** C-Fos levels were elevated in the brain from HDM-sensitized mice, assessed by immunoblotting and immunohistochemistry of paraffin section (brown dots, prominent in the periventricular thalamus). **B**–**D** Chymase, Iba-1 and GFAP expression measured by immunoblotting of brain homogenates. GAPDH was used to normalize potential variation in protein loading on gels. Chymase and GFAP protein were increased in the whole brains of HDM-treated mice. *p < 0.05, **p < 0.01, Mann–Whitney

To identify and characterize the changes in the brain by chronic nasal HDM administration, which might be responsible for the observed behavioral changes, we performed western blotting and immunohistochemistry of brain tissues. Significant increases in the expression of c-Fos and chymase proteins were observed in the brains from mice that received 6 weeks of chronic intranasal HDM compared with PBS control mice (Fig. 8A, B). Iba-1, a marker of microglial activation, was not different between control and HDM groups (Fig. 8C). Glial Fibrillary Acidic Protein (GFAP), a marker of astroglial injury, was elevated in the brains from HDM-sensitized mice (Fig. 8D), indicating an involvement of multiple cell types in the brain. To determine whether the observed behavior changes are related to neuronal injury, we analyzed the cleavage of caspase-3 by western blotting to detect apoptosis in the brain. We did not observe an increased in caspase-3 cleavage from any brain digest samples, demonstrating a lack of apoptotic cell death in the brains of either control or HDM-treated mice (Additional file 2: Fig. S2). This observation was confirmed by TUNEL staining of brain slices (data not shown).

**Fig. 9:**
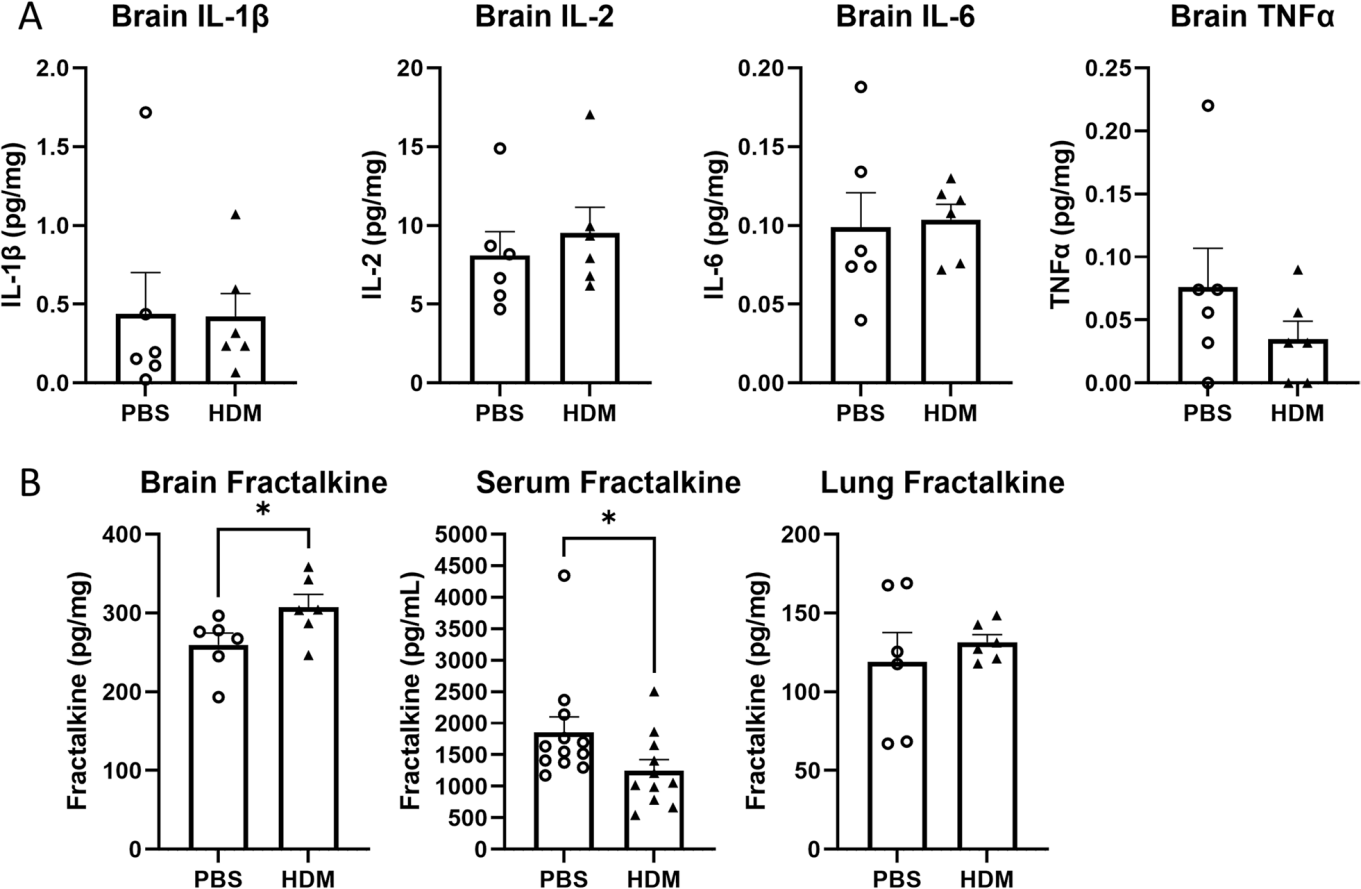
Concentrations of cytokines and chemokines in the whole brain of mice after intranasal sensitization. **A** After completion of behavioral tests, inflammatory cytokines in the whole brain were measured. **B** Fractalkine concentrations in the whole brain, serum and lungs. *p < 0.05, Mann–Whitney

Among brain cytokines measured in whole brain digests, the levels of pro-inflammatory cytokines IL- 1β, IL-2, IL-6 and TNF-α were not significantly different between groups (Fig. 9A). However, brain fractalkine concentrations were significantly elevated in the brains of HDM-treated mice compared with the PBS controls (Fig. 9B, left). Interestingly, serum fractalkine levels were decreased in HDM-treated mice, and no differences were observed in the lungs (Fig. 9B, center and right, respectively).

**Fig. 10:**
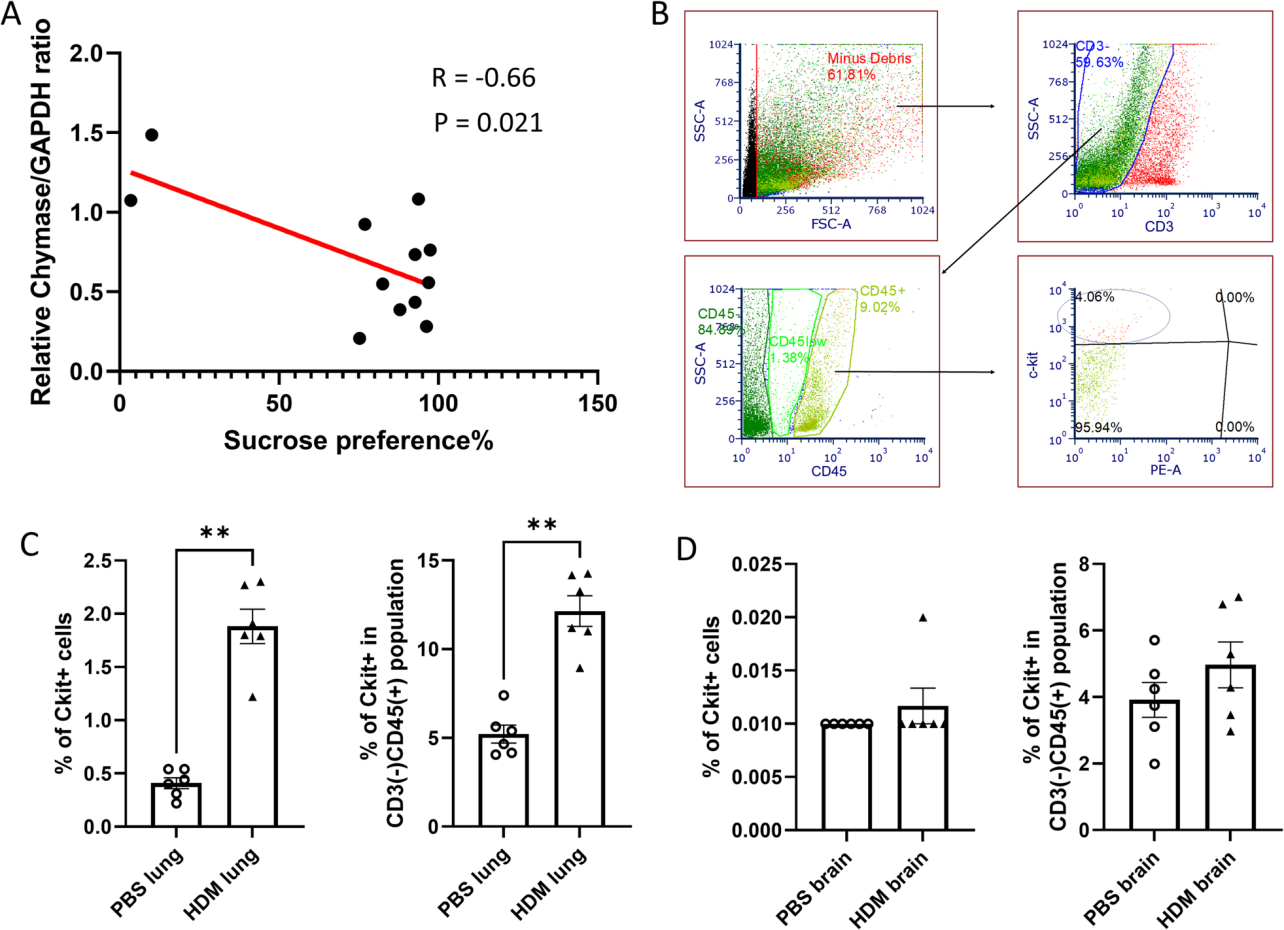
**A** Correlation between chymase expression in the brain and sucrose preference in individual mice. **B** A representative gating used to identify mast cells. **C**, **D** C-kit positive mast cells in the lungs and brain, expressed as % of total singlet cells (left) and in CD3 negative, CD45 positive population (right). **p < 0.01, Mann–Whitney

To further explore a possible association between the observed altered behavior and protein changes in the brain, we performed a univariate analysis. Our observation of sucrose preference test results, in that some mice displayed markedly reduced sucrose preference after HDM sensitization, led us to determine whether there was any association between the degree of anhedonia and protein changes in the brain. Figure 10A shows the linear correlations and the correlation coefficients (*R* = − 0.66) between the relative chymase/GAPDH protein ratios and the sucrose preference, indicating that the relative chymase/GAPDH ratio was negatively correlated with sucrose preference in individual mice. We did not observe significant correlations between the relative c-Fos, Iba-1 or GFAP/GAPDH protein ratios and the sucrose preference (Additional file 3: Fig. S3). Additionally, we performed flow cytometry analysis of brain and lung samples to investigate potential changes in the number of brain mast cells after chronic HDM sensitization. As expected, c-kit positive mast cell numbers were significantly increased in the lungs from HDM-sensitized mice compared with PBS control (Fig. 10B). In the brain, the numbers of c-kit expressing mast cells were extremely low in total singlet cells (Fig. 10C, left) and even in CD3 (T cell marker) negative, CD45 leucocyte common antigen-expressing cell populations (Fig. 10C, right), and we did not observe significant differences between groups.

## Discussion

Chronic non-septic systemic inflammation is known to be associated with neurodegeneration [28]. Allergic asthma is a common chronic inflammatory condition, and it may cause alterations in the immune system, including the brain, which could lead to behavioral changes. In the present study, we demonstrated that after 6 weeks of chronic intranasal HDM administration, mice exhibited altered spatial memory and depression-like behaviors. Their brains showed elevated levels of GFAP, c-Fos and chymase proteins, indicative of astroglial injury, elevated neuronal activity and mast cell activation. To our knowledge, this is the first study reporting behavioral changes utilizing a battery of behavioral tests after chronic nasal HDM sensitization in mice.

HDM is the major source of indoor allergens causing allergic lung inflammation and asthma in humans. It has been widely used in an established mouse asthma model that closely resembles human allergic asthma. Previously, after intratracheal HDM sensitization in A/J mice, reduced motor activity that persisted for only one day was observed in the elevated zero maze [29] that was used to test anxiety-like behaviors. In these prior studies, HDM sensitization was performed once a week for 3 weeks, totaling three times, and other behavioral testing was conducted after fear conditioning by CO_2_ inhalation: therefore, the experimental paradigm and duration of HDM sensitization are both very different from the current study. Others reported aversion to an ovalbumin-associated chamber in ovalbumin sensitized mice [30]. The duration and type of allergen exposure were also different from our study, and their focus was on avoidance behavior that was assessed by a single behavior test performed 24 h after the last ovalbumin challenge. Of note, our behavior experiments were conducted 2–3 days after each HDM sensitization to minimize the potential effect of anesthesia and handling on mice behaviors.

Developmental asthma and adult behavior has also been studied in mice. For example, sensitization with HDM in combination with repeated inhaled aerosolized methacholine challenge during development led to altered anxiety-related behavior with a higher serotonin transporter gene expression in the brainstem in mice [31]. Maternal asthma induced by ovalbumin and behavioral assessments of offspring revealed autism spectrum disorders-like behavioral outcomes in mice [32]. These studies focused on developmental effects of allergic lung inflammation as opposed to the current study focused on adult mice. In addition, these prior studies measured several genes involved with neurotransmitter expressions including serotonin transporter gene, serotonin receptor 1a gene, corticotropin releasing hormone receptor 1 gene and the glucocorticoid receptor gene, but did not measure markers for neuroinflammation in the brain. In the present study, in addition to measuring a series of neurobehavioral outcomes after HDM sensitization introduced to adult mice, we attempted to elucidate the underlying mechanisms by analyzing cytokines in the brain and brain proteins associated with immune cell activation.

Cytokine-mediated communication between the immune system and the brain are implicated in the pathophysiology of mood and behavioral changes [33]. In particular, IL-1β, IL-2, IL-6, TNF-α and IFN-γ are reported to be involved in cytokine-induced depression. For example, IL-6 concentrations have been shown to be increased in patients with depression [34]. An association between IL-2, IL-6, IL-8, TNF-α and VEGF levels and the severity of depression have been reported [35]. Additionally, IL-5 and IL-17 production by T-cells from allergic asthma patients are reported to be positively associated with the severity of major depressive disorders [36]. In the current study, the amount of IL-1β, IL-2, IL-4, IL-5, IL-13 and IFN-γ in the serum of HDM-sensitized mice were significantly higher than PBS control mice, demonstrating that chronic nasal HDM administration leads to systemic increases in these pro-inflammatory and Th2-related cytokines that are potentially associated with depression. The precise mechanisms of behavioral changes induced by systemic increases in cytokines are not fully understood, but a potential proposed mechanism is the blood brain barrier (BBB) disruption that leads to inflammation in the central nervous system: an important factor contributing to neurologic impairment demonstrated by multiple investigators. For example, neuroinflammation and the associated cognitive decline in rats after open tibia fracture surgery [17] and in various rodent model of sepsis has been studied [37, 38]. It was reported that the systemic administration of LPS induced cognitive impairment which was accompanied by neuronal loss, neuroinflammation with microglial activation and damage to the BBB [37, 39]. Therefore, initially we hypothesized that after the development of chronic allergic lung inflammation by long-term administration of intranasal HDM, mice would exhibit increased serum IL-6 and TNF-α concentrations, which had been observed in other systemic inflammation models. We anticipated that these changes in serum IL-6 and TNF-α would lead to BBB disruption [40–42], which would allow for communication of peripheral inflammation to central inflammation leading to neurobehavioral changes. However, we did not observe significant increases in serum IL-6 or TNF-α concentrations, but instead IL-1β levels were significantly increased in the serum from HDM-sensitized mice. However, our serum cytokine measurements were performed at a single time point 10 weeks after the start of sensitization meaning that we may have not detected a change occurring at earlier time points. While TNF-α targets caveolae-mediated transcellular processes of the BBB, IL-1β affects paracellular barriers leading to BBB dysfunction [43]. Serum VEGF concentrations which have been reported to be higher in asthmatic patients and also reported as a biomarker for asthma exacerbation [44], was previously shown to be positively correlated with severity of depression symptoms in asthmatics [45]. VEGF was also reported to enhance the permeability of BBB [46, 47], and it was significantly increased in the serum of HDM-sensitized mice. Taken together, our observed behavioral alterations in HDM-sensitized mice might be attributable to BBB disruption. We also investigated whether the observed behavioral changes were due to neuronal cell death. However, brain samples from HDM-sensitized mice did not show any signs of apoptosis as assessed by cleavage of caspase-3 or TUNEL staining.

Inflammatory cytokines, including L-1β, IL-2, IL-6 and TNF-α, were not significantly increased in the brains of HDM-sensitized mice. Although it is possible that our whole brain sampling at a single later time point may have not detected a transient or site-specific change in cytokine concentrations that was previously reported in a murine endotoxemia model [48]. Our findings are different from previously reported increases in these cytokines in models of infection- or injury-induced behavioral changes, suggesting that the observed behavior changes in this allergic lung inflammation model might not be due to the same mechanisms. Interestingly, only the levels of fractalkine in the whole brain were significantly increased in HDM-sensitized mice. The observed increase was not due to a systemic increase, because serum fractalkine levels were reduced in HDM-sensitized mice. Fractalkine (CX3CL1) is a unique chemokine that can act as either a soluble or membrane-bound mediator, and signals through the G protein-coupled chemokine receptor CX3CR1. In the central nervous system, fractalkine is found mainly in neurons and astrocytes, and its receptor CX3CR1 is expressed on microglia [49, 50]. Fractalkine is reported to be increased after traumatic brain injury [51]. Additionally, fractalkine expression is noted on astrocytes upon inflammatory stimulations while CX3CR1 expression on parenchymal microglia regulates immune responses upon neuroinflammation. Endogenous fractalkine is thought to act as an anti-inflammatory chemokine in the brain through suppressing microglial activation and subsequent inhibition of TNF-α secretion by microglia [52]. Further study examining CX3CR1 expression in microglia after HDM sensitization would be an important future direction.

Based on the key roles of mast cells in the allergic asthmatic response through the secretion of mediators with pro-inflammatory and airway constrictive effects, and the well-known presence of mast cells in the brain, we sought to investigate whether mast cells in the brain are activated during HDM-induced allergic lung inflammation. Our western blot analysis using brain homogenates demonstrated that the c-Fos protein, which is an indirect marker of neuronal activity and elevated upon mast cell activation, was increased in the brains from mice that received chronic 6 weeks of intranasal HDM. Chymase, a serine protease in mast cells, were also increased in the HDM group compared to the intranasal PBS control group. To further explore the role of other immune cells that are potentially activated during allergic lung inflammation and can play a role in brain inflammation, we measured expression of Iba-1 protein, a microglial marker whose expression is generally higher in activated microglia [53]. Microglia are a type of macrophage in the brain whose increase in number is associated with brain inflammation. In the current study, there was no difference in Iba-1 expression in the brain digest of HDM-treated mice and control mice. Emerging evidence suggests that astrocytes play important roles in influencing brain function and pathology in mood disorders [54], and Glial Fibrillary Acidic Protein (GFAP) constitutes the major part of the cytoskeleton of astrocytes and is a biomarker for astroglial injury. The levels of GFAP are reported to be increased in traumatic brain injury [55], gliomas [56], aging and neurologic disorders including multiple sclerosis [57]. Therefore, GFAP expression in the brain was examined to accesses the activation of astrocytes. We found that GFAP expression was significantly increased after chronic HDM treatment compared to the PBS control. These findings suggest correlation between mast cell and astrocyte activation and HDM-induced behavioral changes.

Mast cells are key players in asthma pathophysiology and their accumulation in the lungs of the allergic asthmatic patients are described [58]. This is considered to be due to the recruitment of mast cell progenitors to inflamed lungs [59]. Allergic asthmatic patients with reduced lung function are found to have more mast cell progenitors in the blood circulation [60], potentially increasing the chance of those cells reaching to the brain. Similar to the previous findings, we observed more mast cells in the lungs from HDM-sensitized mice. In the brain, the number of mast cells detected by flow cytometry were very small and the observed increase in HDM-sensitized mice were not statistically significant. Central nervous system mast cell function and their contribution to the modulation of behavior is incompletely understood. Findings of increased EEG delta power, higher anxiety and depression levels were found in mast cell-deficient mice [61, 62]. In a whey protein-induced mouse model of food allergy, increased digging behavior that is thought to reflect repetitive, compulsive-like behaviors, which were associated with increased mast cells in the lateral midbrain and medial hippocampus [63]. Although the number of the mast cells were not reported in their study, inhibition of mast cell degranulation by cromolyn nebulization before ovalbumin nebulization led to inhibition of c-Fos signaling in the brain, and prevented the development of avoidance to ovalbumin in mice [30], indicating that mast cell activation/degranulation was contributing to their observed behavioral change. Therefore, although we did not observe significant change in the numbers of brain mast cells in our study, activation of mast cells observed as increased c-Fos and chymase proteins could contribute to HDM-induced behavioral changes.

We observed altered spatial memory and depression-like behaviors after chronic intranasal HDM sensitization. In the light–dark transition test, HDM-sensitized mice lost a preference for staying in the dark side and crossed the light–dark transition zone more frequently (more exploration), which is typically considered a behavior associated with a reduction in anxiety-like behaviors but could also be a reflection of altered exploratory behavior and spatial memory. For instance, mice generally show less exploration as they learn about the environment, and this behavior is insensitive to anxiolytic treatment, thus does not necessary reflect higher levels of anxiety [64]. In the open field test, mice of the HDM group spent significantly more time in the center after HDM sensitization compared with the center time spent prior to sensitization. This, together with the finding in the light–dark test, suggests a reduced anxiety-like behaviors. The nature of this phenotype warrant further investigation. Previous studies using mast cell-deficient mice or the central injection of the mast cell stabilizer cromolyn, showed decreased number of entries to center square in open field test [62], which is consistent with the hypothesis that our altered behavioral observations may be mediated by brain mast cells. Among the tests performed to assess depression-like behavior, we discovered a difference between mice that underwent HDM sensitization compared to controls in the sucrose preference test and TST. The observed lack of effect in the FST is consistent with a previous report which used a septic mice model [65] and may reflect the different neural mechanisms postulated to regulate performance on the forced swim tests [22]. Although nasal HDM treatment itself may influence the mouse’s senses of smell and taste that might affect their drinking behavior, others using an allergic inflammatory dermatitis mouse model reported decreased sucrose consumption in the sucrose preference test [66], suggesting a link between allergic inflammation and depressive-like behavior. The presence of airway inflammation could potentially be associated with decreased oxygen availability during the TST and FST which may affect test performance. We measured oxygen saturation levels during the last intranasal HDM or PBS administration and found no differences between the groups with the mice at rest (data not shown). However, we cannot exclude the possible influence of airway inflammation on oxygen saturation during intense testing activities due to our inability to measure accurate oxygen saturation on moving animals.

In conclusion, our study demonstrated that allergic lung inflammation in the lung is associated with alterations in brain inflammation and behavioral changes consistent with depression and altered spatial memory. A role for brain mast cell activation and activation of the signaling molecules IL-1β and fractalkine are demonstrated. Further studies investigating the cellular targets and therapeutic interventions to modify lung inflammation may lead to an understanding and prevention of behavioral changes in patients with chronic allergic lung inflammation such as asthma.

## Supplementary Information

**Additional file 1**: **Fig. S1**. Comparison of parameters measured in open field tests. After sensitization treatment, there was no significant differences in the total vertical count, the time spent in the center of the open field, or the total ambulatory distance when comparing means across all mice in the PBS control and HDM groups.

**Additional file 2**: **Fig. S2**. Cleaved caspase-3 fragment was not detected in whole brain homogenates of either PBS control or HDM sensitized mice. Jurkat cell lysates treated with cytochrome c were used as positive control for caspase cleavage.

**Additional file 3: Fig. S3**. Correlation analysis between protein expression in the brain and sucrose preference in individual mice. There was no significant correlation observed between c-Fos, Iba-1 or GFAP protein and the sucrose preference.

## Data Availability

All data are available upon reasonable request to the corresponding author.

## Abbreviations

BAL: Bronchoalveolar lavage
BBB: Blood brain barrier
FCS: Fetal calf serum
FST: Forced swim test
GFAP: Glial fibrillary acidic protein
HDM: House dust mite
IR: Infrared
L/D: Light–dark transition test
NA: Y-maze novel arm
OF: Open field
PBST: PBS containing 0.1% Triton X-100
SA: Y-maze spontaneous alternation
TBST: Tris-buffered saline supplemented with 0.1% Tween 20
TST: Tail suspension test
VEGF: Vascular endothelial growth factor
