# Supplementary figures and images for: Chronic allergic lung inflammation negatively influences neurobehavioral outcomes in mice
Source: J Neuroinflammation. 2022 Aug 31;19:210. doi: 10.1186/s12974-022-02575-y (PMC9429782; doi:10.1186/s12974-022-02575-y)

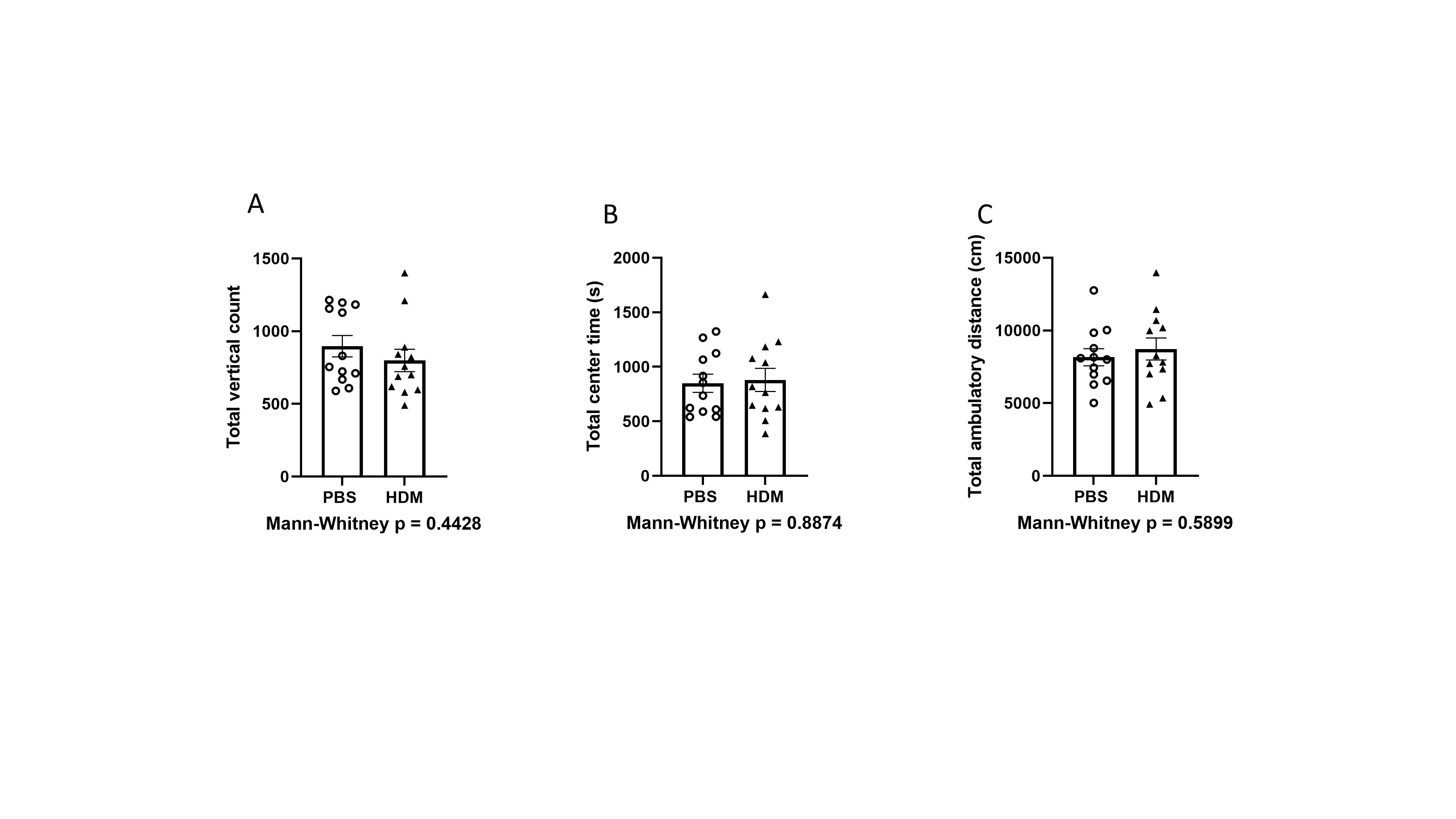

Supplement: Supplementary file 1 — Additional file 1: Fig. S1. Comparison of parameters measured in open field tests. After sensitization treatment, there was no significant differences in the total vertical count, the time spent in the center of the open field, or the total ambulatory distance when comparing means across all mice in the PBS control and HDM groups. [file 12974_2022_2575_MOESM1_ESM.tif]

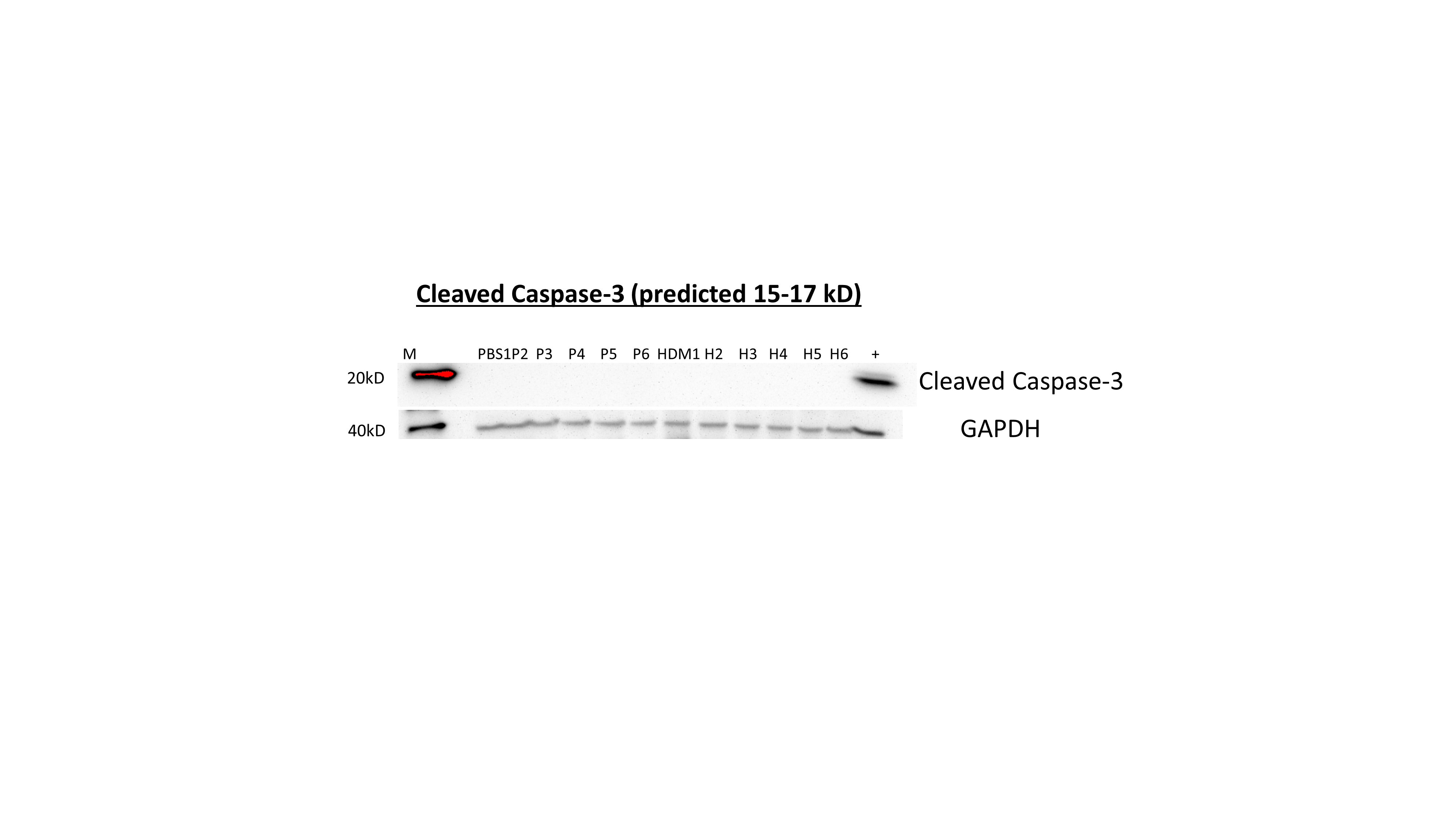

Supplement: Supplementary file 2 — Additional file 2: Fig. S2. Cleaved caspase-3 fragment was not detected in whole brain homogenates of either PBS control or HDM sensitized mice. Jurkat cell lysates treated with cytochrome c were used as positive control for caspase cleavage. [file 12974_2022_2575_MOESM2_ESM.tif]

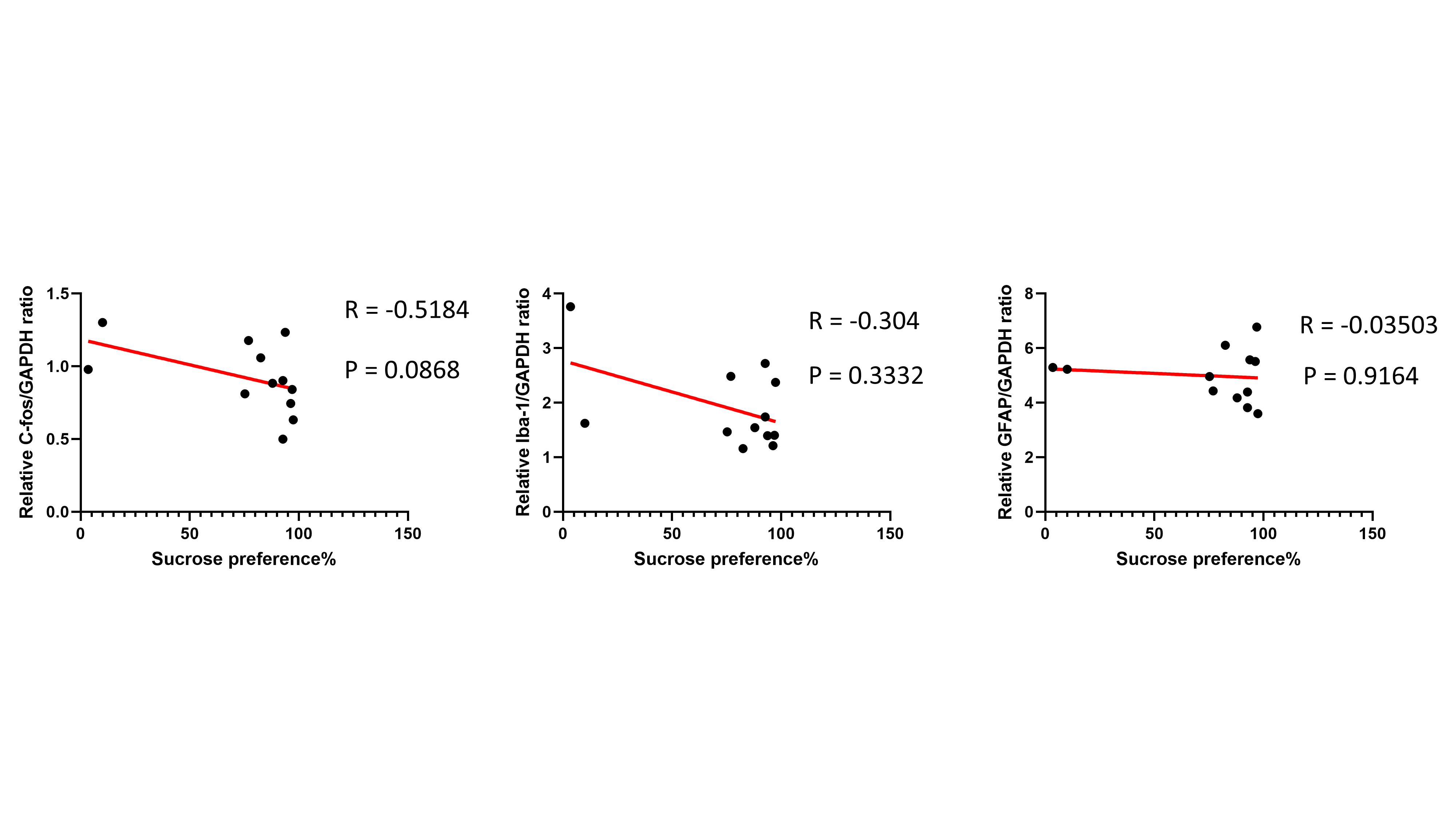

Supplement: Supplementary file 3 — Additional file 3: Fig. S3. Correlation analysis between protein expression in the brain and sucrose preference in individual mice. There was no significant correlation observed between c-Fos, Iba-1 or GFAP protein and the sucrose preference. [file 12974_2022_2575_MOESM3_ESM.tif]
